# Flavones 7,8-DHF, Quercetin, and Apigenin Against Tau Toxicity *via* Activation of TRKB Signaling in ΔK280 Tau_RD_-DsRed SH-SY5Y Cells

**DOI:** 10.3389/fnagi.2021.758895

**Published:** 2021-12-15

**Authors:** Ni-Ni Chiang, Te-Hsien Lin, Yu-Shan Teng, Ying-Chieh Sun, Kuo-Hsuan Chang, Chung-Yin Lin, Hsiu Mei Hsieh-Li, Ming-Tsan Su, Chiung-Mei Chen, Guey-Jen Lee-Chen

**Affiliations:** ^1^Department of Life Science, National Taiwan Normal University, Taipei, Taiwan; ^2^Department of Chemistry, National Taiwan Normal University, Taipei, Taiwan; ^3^Department of Neurology, Chang Gung Memorial Hospital, Chang Gung University School of Medicine, Taoyuan, Taiwan; ^4^Medical Imaging Research Center, Institute for Radiological Research, Chang Gung Memorial Hospital, Chang Gung University, Taoyuan, Taiwan

**Keywords:** Tau, Alzheimer’s disease, quercetin, apigenin, TRKB agonist, 7,8-dihydroxyflavone

## Abstract

Alzheimer’s disease (AD) is a progressive neurodegenerative disease with memory loss and cognitive decline. Neurofibrillary tangles (NFTs) formed by hyperphosphorylated Tau protein are one of the pathological hallmarks of several neurodegenerative diseases including AD. Heat shock protein family B (small) member 1 (HSPB1) is a molecular chaperone that promotes the correct folding of other proteins in response to environmental stress. Nuclear factor erythroid 2-like 2 (NRF2), a redox-regulated transcription factor, is the master regulator of the cellular response to excess reactive oxygen species. Tropomyosin-related kinase B (TRKB) is a membrane-bound receptor that, upon binding brain-derived neurotrophic factor (BDNF), phosphorylates itself to initiate downstream signaling for neuronal survival and axonal growth. In this study, four natural flavones such as 7,8-dihydroxyflavone (7,8-DHF), wogonin, quercetin, and apigenin were evaluated for Tau aggregation inhibitory activity and neuroprotection in SH-SY5Y neuroblastoma. Among the tested flavones, 7,8-DHF, quercetin, and apigenin reduced Tau aggregation, oxidative stress, and caspase-1 activity as well as improved neurite outgrowth in SH-SY5Y cells expressing ΔK280 Tau_RD_-DsRed folding reporter. Treatments with 7,8-DHF, quercetin, and apigenin rescued the reduced HSPB1 and NRF2 and activated TRKB-mediated extracellular signal-regulated kinase (ERK) signaling to upregulate cAMP-response element binding protein (CREB) and its downstream antiapoptotic BCL2 apoptosis regulator (BCL2). Knockdown of TRKB attenuated the neuroprotective effects of these three flavones. Our results suggest 7,8-DHF, quercetin, and apigenin targeting HSPB1, NRF2, and TRKB to reduce Tau aggregation and protect cells against Tau neurotoxicity and may provide new treatment strategies for AD.

## Introduction

Neurodegenerative disorders tauopathies, including the most common Alzheimer’s disease (AD), are characterized by abnormal hyperphosphorylation of microtubule-associated protein Tau that leads to the formation of neurofibrillary tangles (NFTs) and causes gain of toxic function (Iqbal et al., 2005). Tau is mainly expressed in neuronal axons, where it promotes assembly and bundling of microtubules, thereby regulating vesicular transport and apoptosis (Lee et al., 2001). Phosphorylation of Tau has been proposed as the link between oxidative stress, mitochondrial dysfunction, and synaptic failure during early stages of AD (Mondragón-Rodríguez et al., 2013). In retinal ganglion cells of human P301S Tau transgenic mice with early tauopathy, the impairment of tropomyosin-related kinase B (TRKB) signaling is triggered by Tau pathology and mediates the Tau-induced dysfunction of visual response (Mazzaro et al., 2016). In addition, the accumulated asparagine endopeptidase-cleaved N368 Tau N-terminal fragment binds the TRKB receptor on its C terminus and antagonizes neurotrophic signaling to trigger neuronal apoptosis (Xiang et al., 2019).

Tropomyosin-related kinase B, a member of the neurotrophic tyrosine receptor kinase family, is a high-affinity receptor for brain-derived neurotrophic factor (BDNF) (Soppet et al., 1991). Upon BDNF binding, TRKB phosphorylates itself and members of the mitogen-activated protein kinase (MAPK) pathway initiate intracellular signaling cascades (Levine et al., 1996). TRKB is highly expressed in adult hippocampus (Nakagawara et al., 1995), an area of the brain involved in learning and memory. Rapid activation of extracellular signal-regulated kinase (ERK), a member of the MAPK family, by BDNF/TRKB signaling induces phosphorylation of cAMP-response element binding protein (CREB) (Bonni et al., 1999) to stimulate expression of antiapoptotic BCL2 apoptosis regulator (BCL2) (Riccio et al., 1999) for neuronal survival. As a significant risk factor for AD (Coppola et al., 2012), p.A152T Tau variant alters Tau function and toxicity *via* impairing retrograde axonal transport of synaptic vesicles (Butler et al., 2019). As retrograde axonal transport of endosomes mediated by TRKB signaling is essential for dendrite growth of cortical neurons (Zhou et al., 2012), BDNF/TRKB potentiation would be protective in AD.

Structurally, Tau is a prototypical natively unfolded protein (Schweers et al., 1994). Tau binds microtubules through C-terminal highly conserved 18-amino acid repeat domains (Tau_RD_), which reside at the core of paired helical filaments of NFT (Goedert et al., 1989). The Tau_RD_ with the deletion mutation ΔK280 is highly prone to spontaneous aggregation (Khlistunova et al., 2006). Heat shock protein family B (small) member 1 (HSPB1) can prevent pathological misfolding of Tau by altering the conformation of hyperphosphorylated Tau and rescue hyperphosphorylated Tau-mediated cell death (Shimura et al., 2004). Accumulation of misfolded proteins can cause oxidative stress and compromise nuclear factor erythroid 2-like 2 (NRF2) to incur early events in the pathogenesis of AD (Mota et al., 2015). NRF2 activators have therapeutic effects in AD animal models and in cultured human cells that express the pathology of AD (Bahn and Jo, 2019).

Flavones, common in fruits and vegetables, are a class of flavonoids consisting of a benzene A ring condensed with an oxygenated heterocyclic C ring and phenyl B ring. Flavones are plant secondary metabolites with wide range of biological activity including antioxidant, anti-inflammation, and neuroprotective activity (Kumar and Pandey, 2013; Singh et al., 2014). 7,8-dihydroxyflavone (7,8-DHF), the first reported BDNF-mimetic compound, binds with high affinity and specificity to the TRKB receptor (Jang et al., 2010). In addition to improve memory consolidation processes in rats and mice (Bollen et al., 2013), 7,8-DHF activates TRKB signaling to rescue amyloid-beta (Aβ)-induced neurotoxicity and synaptic dysfunction in transgenic mice expressing five familial AD-linked amyloid beta precursor protein (APP) and presenilin 1 (PS1) mutations [5 × familial Alzheimer’s disease (FAD)] (Devi and Ohno, 2012; Zhang et al., 2014). Furthermore, an optimized 7,8-DHF prodrug R13 alleviates Aβ deposition, attenuates the loss of hippocampal synapse, and ameliorates memory deficits in 5 × FAD mice (Chen et al., 2018). We have previously shown neuroprotective effects of 7,8-DHF on neurite length and branch in mouse hippocampal primary neurons under Aβ toxicity and 7,8-DHF-mediated upregulation of neuronal nuclei, p-ERK, and p-CREB in the immunohistochemical staining of mice with oligomeric Aβ injection into hippocampal cornu ammonis area 1 subregion (Fan et al., 2020). In addition, wogonin (5,7-dihydroxy-8-methoxyflavone), a novel inhibitor for mammalian target of rapamycin (mTOR) signaling pathway, strengthens the autophagy to effectively clear Aβ and suppresses Tau protein phosphorylation (Zhu and Wang, 2015). Quercetin (3,3′,4′,5,7-pentahydroxyflavone) decreases β-amyloidosis and tauopathy and protects the cognitive function in APP, PS1, and Tau triple-transgenic AD mouse model (3 × Tg-AD mice) (Sabogal-Guáqueta et al., 2015; Pérez-Corredor et al., 2019). Studies have shown that apigenin (4′,5,7-trihydroxyflavone) ameliorates learning and memory impairment through relieving Aβ burden, suppressing amyloidogenic process, and restoring ERK/CREB/BDNF pathway in APP and PS1 double transgenic AD mice (Zhao et al., 2013). Wogonin, quercetin, and apigenin provide neuroprotection in AD through potentiating HSPB1, NRF2, and TRKB signaling that are not clear. Previously, we have generated human neuroblastoma cells overexpressing ΔK280 Tau_RD_ and used to screen agents that prevent the aggregation and degeneration of cells (Chang et al., 2017). Using this cell model, we demonstrate that 7,8-DHF, quercetin, and apigenin target HSPB1, NRF2, and TRKB to reduce Tau aggregation and protect cells against Tau neurotoxicity, providing new treatment strategies for AD.

## Materials and Methods

### Test Compounds and Biochemical Analysis of Tau Aggregation Inhibition

7,8-dihydroxyflavone, wogonin, quercetin, and apigenin were purchased from Sigma-Aldrich, St. Louis, Mosby, MO, United States. Congo red (Sigma-Aldrich, St. Louis, Mosby, MO, United States), known to bind to the cross β-sheet structure of amyloid fibrils (Sipe and Cohen, 2000), was included for comparison. For biochemical Tau aggregation inhibition test, bacterial expressed proaggregator ΔK280 Tau_RD_ (Gln^244^–Glu^372^ of 441-residue human Tau) was prepared as described (Lin et al., 2020). ΔK280 Tau_RD_ protein (20 μM in final 50 μl) was incubated with tested compounds (5–10 μM in 150 mM sodium chloride (NaCl) and 20 mM tris(hydroxymethyl)aminomethane-hydrochloride (Tris–HCl), pH 8.0) at 37°C for 48 h. Then, thioflavin T (5 μM final concentration; Sigma-Aldrich, St. Louis, Mosby, MO, United States), a dye exhibiting enhanced fluorescence upon binding to diverse types of amyloid fibrils (Biancalana and Koide, 2010), was added and incubated for 25 min at room temperature. The formed aggregates reflected by thioflavin T fluorescence intensity was recorded at excitation 420 nm and emission 485 nm by using the FLx800 Fluorescence Microplate Reader (BioTek Instruments Incorporation, Winooski, VT, United States). Half maximal effective concentration (EC_50_) was calculated using the interpolation method.

### 1,1-Diphenyl-2-Picrylhydrazyl Radical Scavenging Assay

1,1-diphenyl-2-picrylhydrazyl (DPPH) (Sigma-Aldrich, St. Louis, Mosby, MO, United States), a common stable free radical for antioxidant assay (Li et al., 2008), was used to examine the radical scavenging activity of the studied flavones. DPPH solution (100 μM) was prepared in 95% ethanol. After adding test compounds (10–80 μM), the mixture was vortexed for 15 s and allowed to stand for 30 min at room temperature. Subsequently, the mixture was measured spectrophotometrically at 517 nm (Multiskan GO Microplate Spectrophotometer; Thermo Fisher Scientific, Waltham, MA, United States). The free radical scavenging activity was calculated as the percentage of DPPH discoloration using the formula 1 − (absorbance of sample/absorbance of control) × 100%, with EC_50_ calculated using the interpolation method.

### Docking Computation

7,8-dihydroxyflavone is a known potent and selective small-molecule agonist of TRKB (Jang et al., 2010). Using GOLD docking program (Nissink et al., 2002; Verdonk et al., 2003), protein structure of TRKB-d5 domain (pdb code: 1HCF) (Banfield et al., 2001) was utilized to perform docking computation for 7,8-DHF, wogonin, quercetin, and apigenin. In addition to be used to determine the specificity of neurotrophin receptors experimentally (Urfer et al., 1995), the d5 domain was predicted to be the binding site by docking computation (Chitranshi et al., 2015). In docking computations, 10,000, 20,000, 40,000, and 80,000 operations were performed to ensure convergence of the calculated results.

### Cells and Culture

Human neuroblastoma SH-SY5Y-derived ΔK280 Tau_RD_-DsRed cells (Chang et al., 2017) were maintained in Dulbecco’s Modified Eagle Medium (DMEM)/F12 supplemented with 10% fetal bovine serum (FBS) (Thermo Fisher Scientific, Waltham, MA, United States), with 5 μg/ml blasticidin and 100 μg/ml hygromycin (InvivoGen, San Diego, CA, United States) added to the growth medium. Retinoic acid (10 μM; Sigma-Aldrich, St. Louis, Mosby, MO, United States) and doxycycline (2 μg/ml; Sigma-Aldrich, St. Louis, Mosby, MO, United States) were used to induce neuronal differentiation and ΔK280 Tau_RD_-DsRed expression, respectively.

### High Content Analyses of ΔK280 Tau_RD_-DsRed Fluorescence and Oxidative Stress

On day 1, ΔK280 Tau_RD_-DsRed SH-SY5Y cells were seeded into a 96-well plate (2.5 × 10^4^/well), with retinoic acid (10 μM) added to induce neuronal differentiation (Påhlman et al., 1984). On day 2, cells were pretreated with Congo red, 7,8-DHF, wogonin, quercetin, or apigenin (2.5–10 μM) for 8 h, followed by inducing ΔK280 Tau_RD_-DsRed expression with doxycycline. On day 8, cells were stained with Hoechst 33342 (0.1 μg/ml; Sigma-Aldrich, St. Louis, Mosby, MO, United States) for 30 min and cell images were automatically recorded at excitation/emission wavelengths of 543/593 nm (ImageXpress Micro Confocal High-Content Imaging System; Molecular Devices, Sunnyvale, CA, United States) and analyzed (MetaXpress High-Content Image Acquisition and Analysis Software; Molecular Devices, Sunnyvale, CA, United States).

For reactive oxygen species (ROS) measurement, dichloro-dihydro-fluorescein diacetate (DCFH-DA) (10 μM; Invitrogen, Carlsbad, CA, United States), a fluorogenic dye that measures hydroxyl, peroxyl, and other ROS activity within cells (Aranda et al., 2013), was added to the cells on day 8 and incubated at 37°C for 30 min. ROS in cells was measured using the High-Content Imaging System, with excitation/emission wavelengths at 482/536 nm.

### Real-Time PCR Analysis

As described, ΔK280 Tau_RD_-DsRed SH-SY5Y cells were seeded on a 6-well plate (5 × 10^5^/well), with retinoic acid addition on day 1, treated with tested compounds (5 or 10 μM), and induced ΔK280 Tau_RD_-DsRed expression with doxycycline on day 2. On day 8, cells were collected and total RNA was extracted using Trizol reagent (Invitrogen, Carlsbad, CA, United States). The RNA was reverse-transcribed using the SuperScript III Reverse Transcriptase (Invitrogen, Carlsbad, CA, United States). Real-time quantitative PCR was performed using 50 ng complementary DNA (cDNA) with the customized Assays-by-Design probe for DsRed (Chang et al., 2017) and the TaqMan fluorogenic probe for hypoxanthine phosphoribosyltransferase 1 (HPRT1) (4326321E) using the StepOnePlus Real-Time PCR System (Applied Biosystems, Foster City, CA, United States). Fold change was calculated using the formula 2^ΔCt^, ΔC_T_ = C_T_ (HPRT1) − C_T_ (DsRed), in which C_T_ indicates cycle threshold.

### Neurite Outgrowth Analysis

As described, ΔK280 Tau_RD_-DsRed SH-SY5Y cells were seeded on a 24-well plate (5 × 10^4^ cells/well), with induced neuronal differentiation on day 1, treated with test compounds (5 or 10 μM), and induced ΔK280 Tau_RD_-DsRed expression on day 2. After 7 days, the cells were washed with phosphate-buffered saline (PBS) and fixed in 4% paraformaldehyde in PBS for 10 min. After being permeabilized with 0.1% Triton X-100 in PBS for 10 min and blocked with 3% bovine serum albumin in PBS for 20 min, cells were stained with tubulin beta 3 class III (TUBB3) primary antibody (1:1,000; Covance, Princeton, NJ, United States) at 4°C overnight, followed by goat anti-rabbit Alexa Fluor ^®^555 secondary antibody (1:1,000; Invitrogen, Carlsbad, CA, United States) at room temperature for 3 h. After nuclei were stained with 4′,6-diamidino-2-phenylindole (DAPI) (0.1 μg/ml; Sigma-Aldrich, St. Louis, Mosby, MO, United States) for 30 min, neuronal images from at least 60 individual fields (150–250 neurons per field) per experiment were captured at excitation/emission wavelengths of 531/593 nm using the ImageXpress Micro Confocal High-Content Imaging System (Molecular Devices, Sunnyvale, CA, United States). Neurite total length (μm), process (the number of primary neurites defined as the segments originating from the cell body of a neuron), and branch (the number of secondary neurites extending from primary neurites) were analyzed using the MetaXpress Neurite Outgrowth Application Module (Molecular Devices, Sunnyvale, CA, United States). For each sample, around 6,000 cells were analyzed in each of 3 independent experiments.

### Caspase-1, Lactate Dehydrogenase, and Acetylcholinesterase Assays

ΔK280 Tau_RD_-DsRed SH-SY5Y cells were seeded in a 6-well plate (5 × 10^5^/well) and treated with retinoic acid, test compound, and doxycycline as described. On day 8, cells were collected and lysates were prepared by 6 freeze/thaw cycles. After centrifugation to collect supernatant, caspase-1 activity in 50 μg cell extracts was measured using the ICE Fluorometric Assay Kit (BioVision, Milpitas, CA, United States). The mixture was incubated for 2 h at 37°C and caspase-1 activity was measured with excitation/emission wavelengths at 400/505 nm (FLx800 Fluorescence Microplate Reader; BioTek Instruments Incorporation, Winooski, VT, United States). In addition, the collected cells were lysed by sonication. Acetylcholinesterase (AChE) activity in supernatant was determined using AChE Activity Assay Kit (Sigma-Aldrich, St. Louis, Mosby, MO, United States) with 10 μg cell extracts. The mixture was incubated for 2–10 min at room temperature and absorbance at 412 nm was measured using the Multiskan GO Microplate Spectrophotometer Reader (Thermo Fisher Scientific, Waltham, MA, United States). The release of lactate dehydrogenase (LDH) in culture medium was examined by using the LDH Cytotoxicity Assay Kit (Cayman, Ann Arbor, MI, United States). The absorbance was read at 490 nm with the Multiskan GO Microplate Spectrophotometer Reader.

### Ribonucleic Acid Interference

For TRKB knockdown in ΔK280 Tau_RD_-DsRed SH-SY5Y cells, lentivirus carrying TRKB-targeting (TRCN0000002243, TRCN0000002245, and TRCN0000002246) and negative control scrambled (TRC2.Void) short hairpin RNA (shRNA) were obtained from the National RNAi Core Facility, institute of molecular biology/genomic research center, Academia Sinica, Taipei, Taiwan. On day 1, cells were plated on 24-well plates in the presence of retinoic acid as described. On day 2, the cells were infected with lentivirus (3 multiplicity of infection for each shRNA) in medium with polybrene (8 μg/ml; Sigma-Aldrich, St. Louis, Mosby, MO, United States). On day 3, the culture medium was changed and the cells were pretreated with Congo red (10 μM), 7,8-DHF (5 μM), quercetin (5 μM), or apigenin (10 μM) for 8 h, followed by induction of ΔK280 Tau_RD_-DsRed expression. On day 9, the cells were collected for TRKB protein analysis or analyzed for neurite outgrowth as described.

### Protein Blot Analysis

Total proteins from ΔK280 Tau_RD_-DsRed SH-SY5Y cells were prepared using lysis buffer containing 50 mM Tris–HCl pH 8.0, 150 mM NaCl, 1 mM ethylene diamine tetra acetic acid pH 8.0, 1 mM ethylene glycol-bis(β-aminoethyl ether)tetraacetic acid pH 8.0, 0.1% sodium dodecyl sulfate (SDS), 0.5% sodium deoxycholate, 1% Triton X-100, and protease (Sigma-Aldrich, St. Louis, Mosby, MO, United States) and phosphatase (Abcam, Cambridge, MA, United States) inhibitor cocktails. After quantitation using a protein assay kit (Bio-Rad, Hercules, CA, United States), proteins (20 μg) were separated on 10% SDS-polyacrylamide gel electrophoresis and blotted to polyvinylidene difluoride membranes (Sigma-Aldrich, St. Louis, Mosby, MO, United States) by reverse electrophoresis. After blocking, the membrane was probed with antibody against DsRed (1:500; BioVision #3994, Milpitas, CA, United States); HSPB1 (1:500; Abcam #ab137748, Cambridge, MA, United States); NRF2 (1:500; Santa Cruz Biotechnology, Santa Cruz, CA, United States #sc-365949); TRKB (1:500; Cell Signaling, Danvers, MA, United States #4603); p-TRKB (Y817) (1:500; Millipore #ABN1381, Billerica, MA, United States); ERK (1:500; Cell Signaling, Danvers, MA, United States #9102); p-ERK (T202/Y204) (1:500; Cell Signaling, Danvers, MA, United States #9101); CREB (1:1,000; Santa Cruz Biotechnology, Santa Cruz, CA, United States #sc-186); p-CREB (S133) (1:1,000; Millipore #06-519, Billerica, MA, United States); BCL2 (1:500; Santa Cruz Biotechnology, Santa Cruz, CA, United States #sc-7382), BCL2-associated X protein (BAX) (1:500; Cell Signaling, Danvers, MA, United States #2772); or glyceraldehyde-3-phosphate dehydrogenase (GAPDH) (as a loading control) (1:1,000; MDBio #30000002, Taipei, Taiwan). The immune complexes were detected by horseradish peroxidase-conjugated goat antimouse (#GTX213111-01) or goat antirabbit (#GTX213110-01) immunoglobulin G (IgG) antibody (1:5,000, GeneTex Incorporation, Irvine, CA, United States) and chemiluminescent substrate (Millipore, Billerica, MA, United States).

### Statistical Analysis

Data are presented as mean ± SD. 3 independent tests in 2 or 3 biological replicates were performed in each experiment. Differences between groups were evaluated using the two-tailed Student’s *t*-test or the one-way ANOVA with the *post hoc* Tukey’s test where appropriate. *p* < 0.05 indicate a statistically significant difference.

## Results

### Tested Flavones, Radical Scavenging, and Biochemical Tau Aggregation Inhibition

**FIGURE 1:**
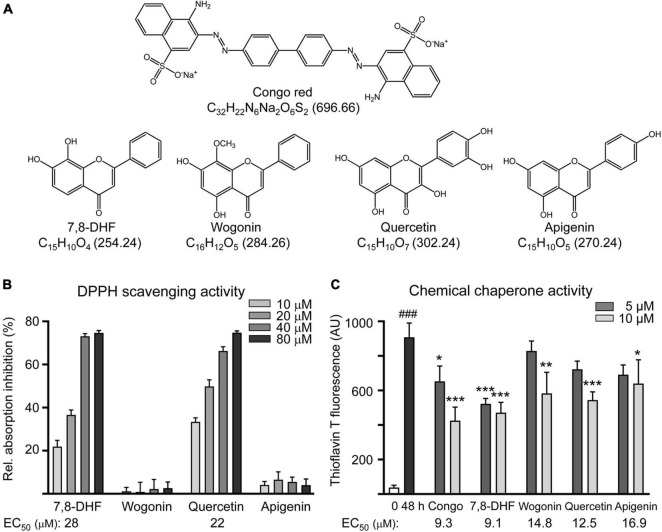
Tested compounds. **(A)** Structure, formula, and molecular weight of Congo red (as a positive control), 7,8-dihydroxyflavone (7,8-DHF), wogonin, quercetin, and apigenin. **(B)** Radical-scavenging activity of tested flavones (10–80 μM) on 1,1-diphenyl-2-picrylhydrazyl (DPPH) (*n* = 3). Shown below are the EC_50_ values. **(C)** ΔK280 Tau_RD_ aggregation inhibition of tested compounds (5–10 μM) by the thioflavin T assay (*n* = 3). *p-*values: comparisons between 0-h and 48-h incubation (^###^*p* < 0.001) or between with and without compound addition (*p < 0.05, **p < 0.01, and ***p < 0.001) (one-way ANOVA with the *post hoc* Tukey’s test). Shown below are the EC_50_ values.

Natural flavones such as 7,8-DHF, wogonin, quercetin, and apigenin (Figure 1A) were examined. Oxidative stress has been recognized as a contributing factor to the progression of AD and preventing oxidative stress is considered as a treatment approach for AD (Poprac et al., 2017). Therefore, the radical scavenging activity of the three flavones (10–80 μM) was first examined. While wogonin and apigenin displayed no radical scavenging activity, 7,8-DHF and quercetin had EC_50_ values of 24 and 25 μM, respectively (Figure 1B).

The inhibition of Tau aggregation was measured by measuring thioflavin T binding to *Escherichia coli*-derived 14.7 kDa ΔK280 Tau_RD_ protein using Congo red as a control. EC_50_ values of Congo red, 7,8-DHF, wogonin, quercetin, and apigenin for Tau aggregation inhibition were: 9.3, 9.1, 14.8, 12.5, and 16.9 μM, respectively (Figure 1C).

### Tropomyosin-Related Kinase B Binding Prediction

**FIGURE 2:**
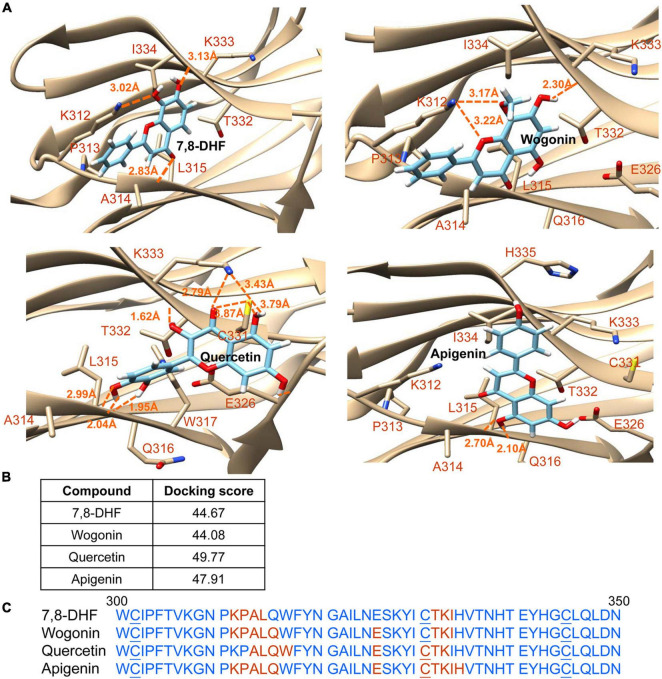
Docking computations of 7,8-DHF, wogonin, quercetin, and apigenin. **(A)** The docking conformations of tested flavones binding to d5 domain (the second immunoglobulin-like domain, 250–340 amino acid residues) of extracellular domain of tropomyosin-related kinase B (TRKB) receptor. The TRKB-d5 domain (ribbon structures) is colored in beige and the wire-frame structures denote the compounds. The labeled amino acids were within 10 Å radii of examined compounds. Carbon, oxygen, hydrogen, and nitrogen atoms of compounds or side chains of surrounding amino acids are shown in light blue, red, white, and blue, respectively. The dotted orange lines indicate hydrogen bond interactions between compounds and proteins. **(B)** The docking scores of 7,8-DHF, wogonin, quercetin, and apigenin calculated by the GOLD program. **(C)** Amino acid residues 301–350 of d5 domain. The amino acids within 10 Å radii of examined compounds are colored in red; the cysteines involved in disulfide linkage are underlined.

The strengths and conformation of wogonin, quercetin, and apigenin binding with neurotrophin-binding domain d5 of TRKB were calculated using 7,8-DHF as a control. As shown in Figure 2, the computations predicted docking scores of 44.67, 44.08, 49.77, and 47.91 for 7,8-DHF, wogonin, quercetin, and apigenin, respectively. Quercetin had the top interacting docking score with TRKB receptor among the 3 flavones examined.

### Cellular Tau Aggregation Inhibition and Oxidative Stress Reduction in ΔK280 Tau_RD_-DsRed-Expressing SH-SY5Y Cells

**FIGURE 3:**
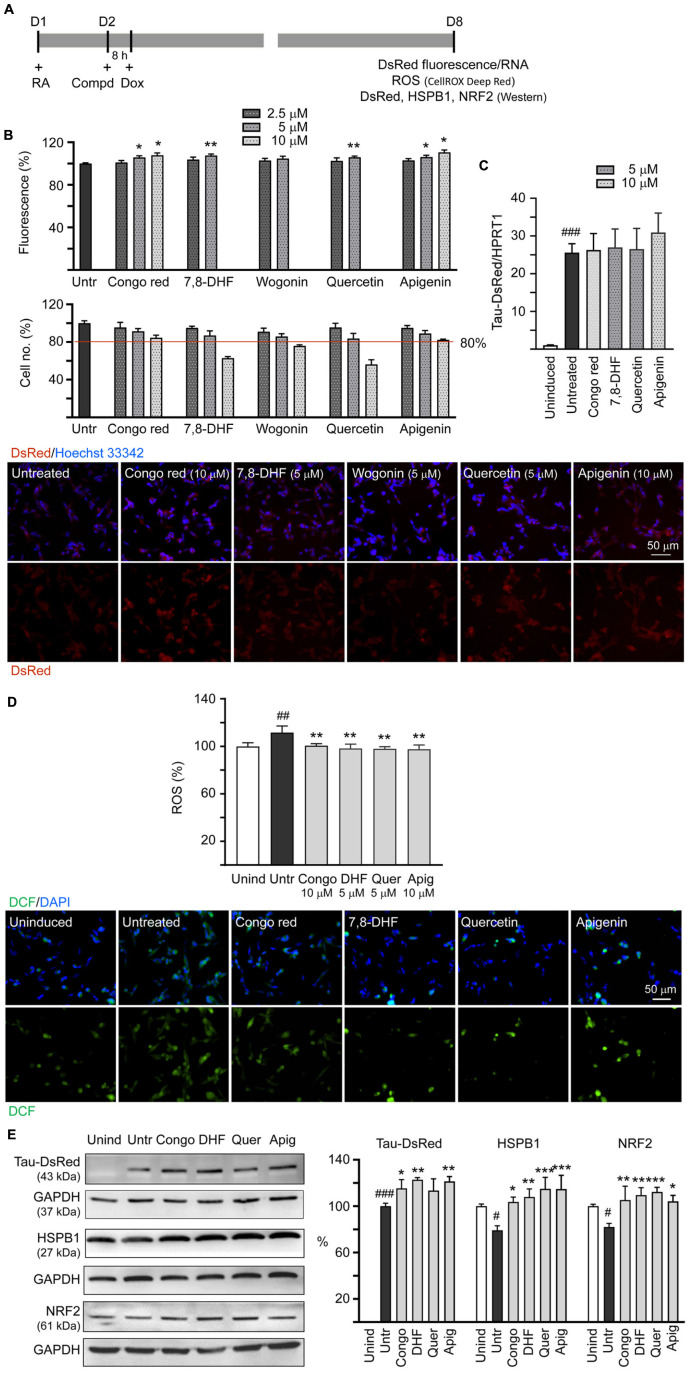
Tau aggregation inhibition and reactive oxygen species (ROS) reduction in SH-SY5Y cells expressing ΔK280 Tau_RD_-DsRed. **(A)** Experimental flowchart. On day 1, cells were plated with retinoic acid (RA) (10 μM) added to the culture medium. On day 2, Congo red (as a positive control), 7,8-DHF, wogonin, quercetin, or apigenin was added to the cells for 8 h, followed by inducing ΔK280 Tau_RD_-DsRed expression with doxycycline (Dox) (2 μg/ml) for 6 days. On day 8, ΔK280 Tau_RD_-DsRed fluorescence/RNA, ROS dichlorofluorescein (DCF stain), as well as DsRed, heat shock protein family B (small) member 1 (HSPB1), and nuclear factor erythroid 2-like 2 (NRF2) protein levels were measured. **(B)** Top: Assessment of DsRed fluorescence with Congo red, 7,8-DHF, wogonin, quercetin, or apigenin (2.5–10 μM) treatment and cell number (obtained by counting Hoechst 33342 stained cell nuclei) analyzed (*n* = 3). The relative DsRed fluorescence/cell number of untreated cells (Untr) was normalized as 100%. *p-*values: comparisons between with and without compound addition (*p < 0.05, **p < 0.01; two-tailed Student’s *t*-test). Bottom: Fluorescent images of ΔK280 Tau_RD_-DsRed cells with or without Congo red (10 μM), 7,8-DHF (5 μM), wogonin (5 μM), quercetin (5 μM), or apigenin (10 μM) treatment. Nuclei were counterstained with Hoechst 33342 (blue). **(C)** Real-time PCR analysis of Tau_RD_-DsRed RNA in SH-SY5Y cells uninduced, untreated, or treated with 5 or 10 μM compound (*n* = 3). HPRT1 was used as an endogenous control to normalize between samples. **(D)** Top: Quantitation of ROS (*n* = 3) and images of DCF stain (green) of ΔK280 Tau_RD_-DsRed cells uninduced, untreated, or treated with Congo red (10 μM), 7,8-DHF (5 μM), quercetin (5 μM), or apigenin (10 μM). The relative ROS of uninduced cells was normalized as 100%. Bottom: Images of DCF (green) assay of ΔK280 Tau_RD_-DsRed cells uninduced, untreated, or treated with Congo red (10 μM), 7,8-DHF (5 μM), quercetin (5 μM), or apigenin (10 μM). Nuclei were counterstained with 4′,6-diamidino-2-phenylindole (DAPI) (blue). **(E)** Immunoblot analysis of Tau_RD_-DsRed, HSPB1, and NRF2 proteins in SH-SY5Y cells uninduced, untreated, or treated with 5 or 10 μM compound (*n* = 3). Glyceraldehyde-3-phosphate dehydrogenase (GAPDH) was used as a loading control. To normalize, the HSPB1 or NRF2 expression level in uninduced (Unind) cells was set at 100%. For Tau_RD_-DsRed, the soluble level in untreated cells (Untr) cells was set at 100%. **(D,E)** *p*-values: comparisons between untreated vs. uninduced cells (^#^*p* < 0.05, ^##^*p* < 0.01, ^###^*p* < 0.001) or compound-treated vs. untreated cells (*p < 0.05, **p < 0.01, ***p < 0.001) (one-way ANOVA with the *post hoc* Tukey’s test).

Neuronal differentiated human SH-SY5Y cells were used to evaluate the effects of tested flavones on ΔK280 Tau_RD_ aggregation inhibition, ROS reduction, and HSPB1 and NRF2 expression changes (Figure 3A). In these cells, the misfolded ΔK280 Tau_RD_ adversely affected the folding of fused DsRed to decrease DsRed fluorescence (Chang et al., 2017). DsRed fluorescence was measured in wells containing at least 80% cells remained compared to untreated cells. Congo red, known to attenuate amyloid-like aggregates of neuronal Tau induced by formaldehyde (Nie et al., 2007), was included for comparison. As shown in Figure 3B, after normalization of DsRed fluorescence with cell number counted, treatment with Congo red at 5–10 μM (106–108%; *p* = 0.018–0.017), 7,8-DHF at 5 μM (108%; *p* = 0.004), quercetin at 5 μM (106%; *p* = 0.004), or apigenin at 5–10 μM (106–110%; *p* = 0.016–0.010) increased the DsRed fluorescence intensity significantly compared with untreated cells (100%), but treatment with wogonin did not increase the DsRed fluorescence intensity. Under circumstance of over 80% cells remained, treatment of 10 μM Congo red, 5 μM 7,8-DHF, 5 μM quercetin, or 10 μM apigenin did not altered Tau-DsRed RNA level significantly (26.3–30.9-folds, *p* > 0.05) compared with untreated cells (25.6-fold) (Figure 3C). Given that wogonin did not show aggregation-inhibiting and free radical scavenging effects, only Congo red, apigenin at 10 μM and 7,8-DHF, quercetin at 5 μM were adopted in the subsequent experiments.

Misfolded Tau may increase the production of ROS (Cente et al., 2006). Thus, we evaluated antioxidative effects of 7,8-DHF, quercetin, and apigenin using DCFH-DA. Induced ΔK280 Tau_RD_-DsRed expression elevated the ROS level in these cells (112%; *p* = 0.005) and Congo red (10 μM), 7,8-DHF (5 μM), quercetin (5 μM), or apigenin (10 μM) effectively reduced the ROS level associated with ΔK280 Tau_RD_ overexpression (from 112 to 101–98%; *p* = 0.008–0.001) (Figure 3D).

We also examined if Congo red, 7,8-DHF, quercetin, and apigenin upregulate HSPB1 and NRF2 expression in ΔK280 Tau_RD_-DsRed SH-SY5Y cells. As shown in Figure 3E, addition of Congo red (10 μM), 7,8-DHF (5 μM), quercetin (5 μM), or apigenin (10 μM) increased soluble ΔK280 Tau_RD_-DsRed (from 100 to 113–123%; *p* = 0.098–0.003) and HSPB1 (from 79 to 104–115%; *p* = 0.016– < 0.001) levels. In addition, addition of Congo red or tested flavones (5 or 10 μM) increased NRF2 protein level (from 82 to 104–112%; *p* = 0.010– < 0.001), which is essential for defense against ROS.

### Caspase-1, AChE Reduction, and Neurite Outgrowth Promotion in ΔK280 Tau_RD_-DsRed-Expressing SH-SY5Y Cells

**FIGURE 4:**
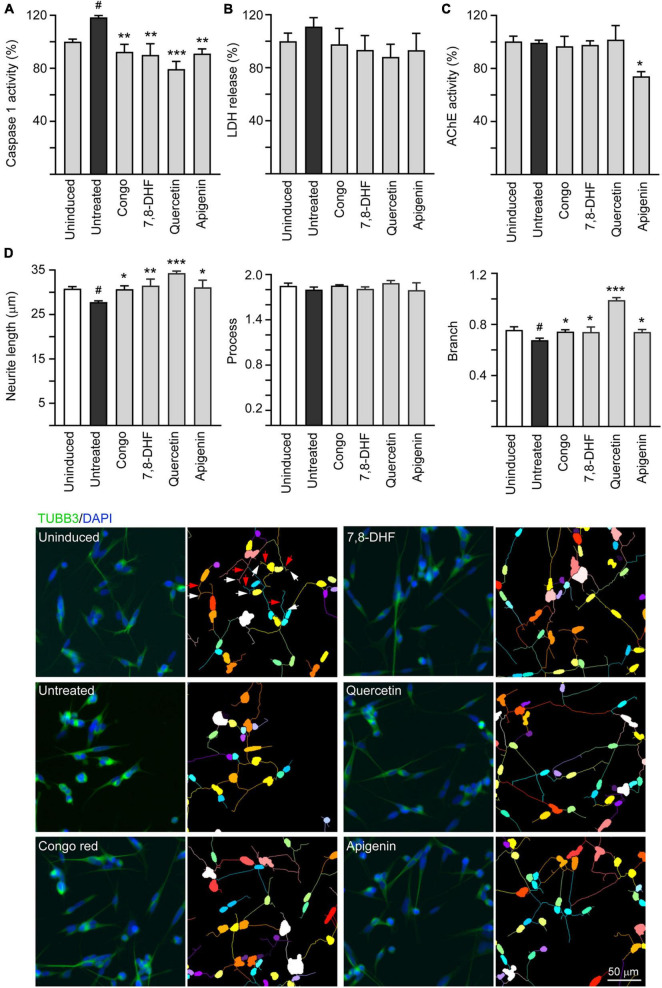
Caspase-1, acetylcholinesterase (AChE) reduction, and neurite outgrowth promotion of 7,8-DHF, quercetin, and apigenin in SH-SY5Y cells expressing ΔK280 Tau_RD_-DsRed. As described, cells were seeded with RA, treated with Congo red (10 μM), 7,8-DHF (5 μM), quercetin (5 μM), or apigenin (10 μM), and induced ΔK280 Tau_RD_-DsRed expression with doxycycline for 6 days. On day 8, **(A)** caspase-1 activity, **(B)** lactate dehydrogenase (LDH) release, **(C)** AChE activity, and **(D)** neurite length, process, and branch were analyzed (*n* = 3). To normalize, the relative caspase-1, LDH release, or AChE activity of uninduced cells (Dox -) was set as 100%. Shown on the bottom half of panel **(D)**, there were images of TUBB3 (green)-stained cells, with nuclei counterstained with DAPI (blue) and segmented images with multicolored mask to assign each outgrowth to a cell body for neurite outgrowth quantification. Process and branches in uninduced cells were marked with red and white arrows, respectively. *p*-values: comparisons between induced vs. uninduced cells (^#^*p* < 0.05) or compound-treated vs. untreated cells (*p < 0.05, **p < 0.01, ***p < 0.001) (one-way ANOVA with the *post hoc* Tukey’s test).

In the brains of patients with AD, cholinergic neurons were selectively impaired (Davies and Maloney, 1976) and AChE inhibitors have been used as standard treatment of AD (Citron, 2010). In addition, caspase-1 activation is associated with brain pathology of AD (Heneka et al., 2013) and caspase-1 inhibition alleviates cognitive impairment and neuropathology (Flores et al., 2018) in AD mouse models. Therefore, caspase-1 and AChE activities in compound-treated cells were also evaluated. The overexpression of ΔK280 Tau_RD_ significantly increased caspase-1 activity (118%; *p* = 0.020) and treatment with Congo red, 7,8-DHF, quercetin, and apigenin (5 or 10 μM) reduced the caspase-1 activity (from 118 to 92–79%; *p* = 0.002– < 0.001) (Figure 4A). Similar changing trend of LDH release was also observed (from 111 to 98–88%), although not significant (*p* > 0.05) (Figure 4B). In SH-SY5Y cells, ΔK280 Tau_RD_ overexpression did not increased AChE activity, but treatment with apigenin (10 μM) reduced AChE activity (from 99 to 74%; *p* = 0.016) compared to no treatment (Figure 4C).

Tau is a microtubule-associated protein that plays a role in mediating neurite outgrowth (Johnson and Stoothoff, 2004). Calpain-cleaved neurotoxic Tau_45–230_ fragment modifies the composition of the neuronal cytoskeleton and impairs neurite elongation in neurons undergoing degeneration (Afreen and Ferreira, 2019). The neurite outgrowth-promoting effect of tested compounds including total neurite length, number of processes, and number of branch points was evaluated. As shown in Figure 4C, overexpression of ΔK280 Tau_RD_ significantly decreased neurite length (from 30.8 to 27.8 μm; *p* = 0.026) and branch (from 0.76 to 0.67; *p* = 0.010). Treatment with Congo red (10 μM), 7,8-DHF (5 μM), quercetin (5 μM), and apigenin (10 μM) rescued the impairment of neurite length (from 27.8 to 30.7–34.3 μm; *p* = 0.035– < 0.001) and branch (from 0.67 to 0.74–0.99; *p* = 0.034– < 0.001).

### Molecular Targets of 7,8-DHF, Quercetin, and Apigenin in ΔK280 Tau_RD_-DsRed-Expressing SH-SY5Y Cells

**FIGURE 5:**
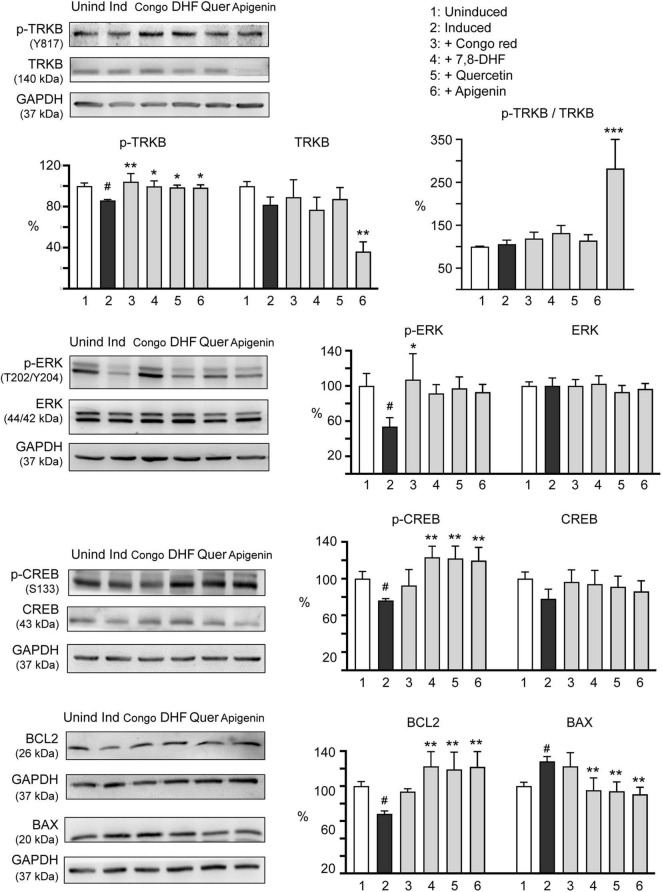
Molecular targets of 7,8-DHF, quercetin, and apigenin in SH-SY5Y cells expressing ΔK280 Tau_RD_-DsRed. Congo red (10 μM) was included as a negative control. Relative TRKB, p-TRKB (Y817), p-TRKB/TRKB, extracellular signal-regulated kinase (ERK), p-ERK (T202/Y204), cAMP-response element binding protein (CREB), p-CREB (S133), BCL2 apoptosis regulator (BCL2), and BCL2-associated X protein (BAX) protein levels were analyzed through immunoblotting using specific antibodies (*n* = 3). GAPDH was used as a loading control. Relative protein levels are shown on the right side of the representative western blot images. To normalize, the relative protein level in uninduced cells (Dox -) was set as 100%. *p*-values: comparisons between induced and uninduced cells (^#^*p* < 0.05) or between treated and untreated cells (*p < 0.05, **p < 0.01, ***p < 0.001) (one-way ANOVA with the *post hoc* Tukey’s test).

Tropomyosin-related kinase B is highly expressed in the hippocampus and plays a critical role in memory processes (Kang et al., 1997). Phosphorylation of TRKB at the most C-terminal tyrosine, Y817, leads to activation of ERK and CREB and transcription of BCL2 to promote cell survival (Hausott et al., 2009). The effects of Congo red (10 μM), 7,8-DHF (5 μM), quercetin (5 μM), and apigenin (10 μM) on expression levels of TRKB, ERK, CREB, and BCL2 in ΔK280 Tau_RD_-DsRed-expressing SH-SY5Y cells were examined (Figure 5). Addition of tested flavones increased p-TRKB (Y817) (from 86 to 99–100%; *p* = 0.039–0.022), p-ERK (T202/Y204) (from 54 to 92–97%; *p* = 0.105–0.052), p-CREB (S133) (from 76 to 120–123%; *p* = 0.003–0.001), and downstream BCL2 (from 68 to 119–123%; *p* = 0.002–0.001) protein levels. In response to the antiapoptotic BCL2 change, addition of tested flavones significantly reduced the expression of proapoptotic BAX (from 128 to 95–91%; *p* = 0.003–0.002). Although Congo red treatment increased p-TRKB (Y817) (104%; *p* = 0.003) and p-ERK (T202/Y204) (107%; *p* = 0.014), changes of p-CREB (S133) (92 vs. 76%), BCL2 (94 vs. 68%), and BAX (122 vs. 128%) were not significant (*p* > 0.05) in ΔK280 Tau_RD_-DsRed-expressing SH-SY5Y cells. Due to reduction of the total amount of TRKB (36%; *p* = 0.001), apigenin induced a significant hyperphosphorylation of TRKB in ΔK280 Tau_RD_-DsRed-expressing SH-SY5Y cells (p-TRKB/TRKB: 282%; *p* < 0.001). The reason for observed reduced TRKB level in apigenin-treated cells is not clear.

### Tropomyosin-Related Kinase B Knockdown in ΔK280 Tau_RD_-DsRed-Expressing SH-SY5Y Cells

**FIGURE 6:**
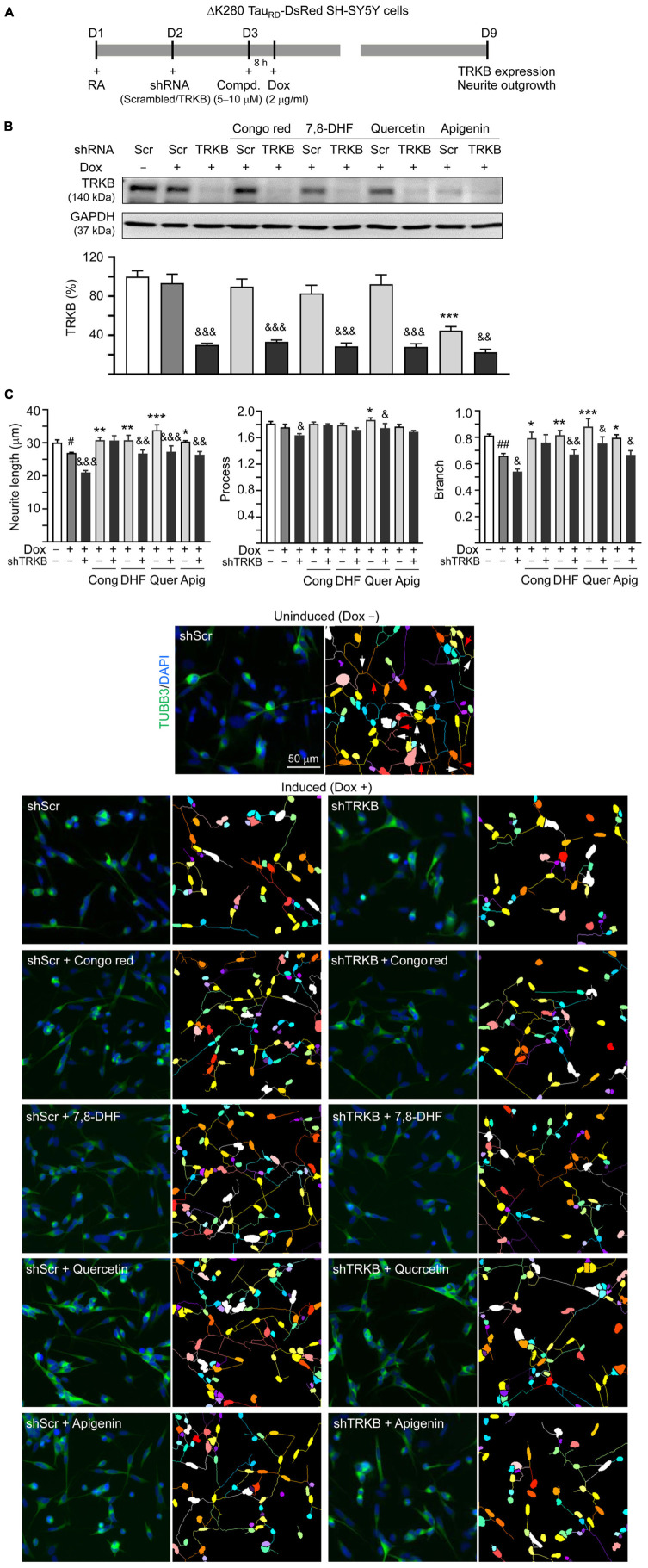
TRKB RNA interference of SH-SY5Y cells expressing ΔK280 Tau_RD_-DsRed. **(A)** Experimental flowchart. On day 1, ΔK280 Tau_RD_-DsRed SH-SY5Y cells were plated with RA (10 μM). On day 2, the cells were infected with lentivirus-expressing TRKB-specific or scrambled shRNA. At 24 h postinfection, Congo red (10 μM), 7,8-DHF (5 μM), quercetin (5 μM), or apigenin (10 μM) was added to the cells for 8 h, followed by induction of ΔK280 Tau_RD_-DsRed expression (Dox, 2 μg/ml) for 6 days. On day 9, the cells were collected for **(B)** TRKB protein (GAPDH as a loading control) and **(C)** neurite outgrowth analyses (*n* = 3). To normalize, the relative TRKB protein of uninduced cells (Dox -) was set as 100%. Shown on the bottom half of panel **(C)**, there were images of TUBB3 (green)-stained cells, with nuclei counterstained with DAPI (blue) and segmented images with multicolored mask to assign each outgrowth to a cell body for neurite outgrowth quantification. Processes and branches in scrambled shRNA-infected uninduced cells were marked with red and white arrows, respectively. *p*-values: comparisons between induced (Dox +) vs. uninduced (Dox -) cells (^#^*p* < 0.05, ^##^*p* < 0.01) between compound-treated vs. untreated cells (*p < 0.05, **p < 0.01, ***p < 0.001) or between TRKB shRNA-treated vs. scrambled shRNA-treated cells (^&^*p* < 0.05, ^&&^*p* < 0.01, ^&&&^*p* < 0.001) (one-way ANOVA with the *post hoc* Tukey’s test).

Since the studied compounds displayed potential to activate TRKB signaling, we knocked down TRKB expression through lentivirus-mediated shRNA targeting in ΔK280 Tau_RD_-DsRed SH-SY5Y cells to examine if TRKB was the therapeutic target of 7,8-DHF, quercetin, and apigenin (Figure 6A). As shown in Figure 6B, no significant change of TRKB level (93 vs. 100%; *p* > 0.05) was observed in scrambled shRNA-infected cells with or without inducing ΔK280 Tau_RD_ expression. While apigenin reduced TRKB level (from 93 to 45%; *p* < 0.001), addition of Congo red, 7,8-DHF, or quercetin did not affect TRKB level in scrambled shRNA-infected cells expressing ΔK280 Tau_RD_ (83–92 vs. 93%; *p* > 0.05). However, TRKB-specific shRNA significantly reduced TRKB in ΔK280 Tau_RD_-DsRed SH-SY5Y cells treated without (from 93 to 30%, *p* < 0.001) or with (from 92–45 to 33–23%, *p* = 0.007– < 0.001) compound.

For neurite outgrowth analysis (Figure 6C), neurite length (from 30.0 to 26.9 μm; *p* = 0.030) and branch (from 0.81 to 0.66; *p* = 0.006) were reduced upon induction of ΔK280 Tau_RD_ expression in scrambled shRNA-infected cells. Congo red, 7,8-DHF, quercetin, or apigenin increased neurite length (from 26.9 to 30.3–33.8 μm; *p* = 0.013– < 0.001) and branch (from 0.66 to 0.79–0.88; *p* = 0.022– < 0.001). In line with TRKB knockdown, TRKB-specific shRNA significantly reduced neurite length and branch without (length: from 26.9 to 21.0 μm, *p* < 0.001; branch: from 0.66 to 0.54, *p* = 0.049) or with (length: from 30.3–33.8 to 26.4–27.6 μm, *p* = 0.003– < 0.001; branch: from 0.81–0.88 to 0.67–0.76, *p* = 0.033–0.009) 7,8-DHF, quercetin, or apigenin addition, but not with Congo red addition (length: 30.7 vs. 30.8 μm, branch: 0.76 vs. 0.79; *p* > 0.05). These results suggested that 7,8-DHF, quercetin, and apigenin improved neurite outgrowth phonotype by upregulating TRKB signaling.

## Discussion

Effective treatments to slow neurodegeneration of AD are still unavailable. BDNF, a well-characterized member of the nerve growth factor family, is expressed in whole brain (Jones and Reichardt, 1990). Binding of BDNF to TRKB receptor activates downstream signaling pathways to promote neuronal growth, survival, and neural plasticity as well as enhance memory formation and storage (Nagahara and Tuszynski, 2011; Lu et al., 2013). The expanding role of BDNF suggests a valuable therapeutic target for AD. However, BDNF has a poor biostability such as the very short plasma half-life and the limited diffusion crossing blood–brain barrier (BBB) (Dittrich et al., 1996). Small compounds that mimic neurotrophic signaling and overcome the pharmacokinetic barrier of BDNF may have greater therapeutic potential (Zeng et al., 2013). In human SH-SY5Y cells expressing proaggregator Tau, we found protective potentials of three natural flavones, 7,8-DHF, quercetin, and apigenin, including reduction of Tau aggregation, ROS and caspase-1, as well as promotion of neurite outgrowth (Figures 3, 4). Similarly, promotion of neurite outgrowth by quercetin in Figure 4 has been reported in pheochromocytoma cell line (Katebi et al., 2019). TRKB silencing counteracted the neuroprotective effects of these flavones against toxicity of proaggregator Tau (Figure 6), demonstrating that the neuroprotective effects of three natural flavones against Tau toxicity were mechanically mediated by increasing p-TRKB (Y817) (Figure 5) to enhance TRKB signaling, in addition to directly interfering with Tau aggregate formation (Figure 1). As knockdown of TRKB is not complete, the added flavones may still exert a modest neuroprotective effect by binding to the remained TRKB, so that the flavone-treated cells with TRKB knockdown still displayed improved neurite outgrowth compared to those without flavone treatment (Figure 6). The mechanism is supported by the high docking scores of the tested compounds binding to the neurotrophin-binding domain d5 of TRKB predicted by the computations (Figure 2). The observed neuroprotective effect under the knockdown of TRKB by these flavones may be also attributed to the increased NRF2 to promote neurite outgrowth (Yang et al., 2015).

Various natural flavones display antioxidant activity (Pietta, 2000). The majority of neurodegenerative diseases are speculated to originate from cumulative oxidative stress caused by deposition of abnormal aggregated proteins (Grimm et al., 2011). There is clear evidence that ROS is highly upregulated in the brain of tauopathies of the patients and ROS also directly promotes Tau modifications (Haque et al., 2019). Compounds with antioxidative potential may directly serve as chemical chaperone to suppress protein aggregates or quench free oxygen radicals. All the three flavones displayed some degree of chemical chaperone activity to enhance the folding and/or stability of proaggregator Tau (Figure 1C). Structural requirements of flavonoids for appreciable radical scavenging activity have been established (Seyoum et al., 2006). Without o-dihydroxy group (catechol structure) in the benzene ring, apigenin has poor antioxidant capacity for scavenging free radicals as assessed using stable radical DPPH (Figure 1B).

An alternative way to decrease cellular ROS is by enhancing antioxidative signaling such as NRF2 pathway. Stabilized following oxidative stress, NRF2 induces the expression of antioxidants as well as cytoprotective genes (Vomund et al., 2017). Reduced nuclear levels of NRF2 are observed in postmortem brains of patients with AD (Ramsey et al., 2007) and NRF2 inducer carnosic acid improves learning and memory in 3 × Tg-AD mice (Lipton et al., 2016). In this study, all the three flavones enhanced the expression of NRF2 (Figure 3E), supporting the role of 7,8-DHF, quercetin, and apigenin in activating the adaptive response to reduce oxidative stress. The NRF2 expression can be upregulated by AKT serine/threonine kinase 1 (AKT), which is one of the downstream proteins of TRKB activation (Yoo et al., 2017). Therefore, the NRF2 in the ΔK280 Tau_RD_ cell model treated with the three flavones may be increased through TRKB activation, at least partially.

Heat-shock proteins such as HSP90, HSP40, and HSPB1 had been speculated to function as regulators of soluble Tau protein levels (Sahara et al., 2007). As all the three flavones enhanced the expression of HSPB1 for chaperoning misfolded Tau (Figure 3E), 7,8-DHF, quercetin, and apigenin may also reduce the Tau-associated ROS through eliminating the misfolded Tau deposits. HSPB1 is transcriptionally upregulated by heat shock transcription factor 1 that can be enhanced by NRF2 (Paul et al., 2018). The HSPB1 expression in the ΔK280 Tau_RD_ cell model treated with the tested compounds may be activated by increased NRF2. However, further studies are required to consolidate the assumption.

Reactive oxygen species-associated neuroinflammation has been well known to be involved in caspase-1 activation and cleavage of proinflammatory cytokines (Howley and Fearnhead, 2008). Oxidative stress may also directly activate caspase-1 involved in inflammasome (Bai et al., 2020). Active caspase-1 induces caspase-6 activation that leads to axonal and neuronal degeneration in human primary central nervous system (CNS) cultures (Kaushal et al., 2015). Both the quercetin and apigenin have been reported to suppress inflammasome activation and downstream effector caspase-1 in human diseases induced by inflammatory responses (Yi, 2018). In this study, 7,8-DHF, quercetin, and apigenin counteract increased caspase-1 activity induced by proaggregator Tau (Figure 4B), which may be attributed to their free radical-scavenging (Figure 1B) or NRF2-enhancing activity (Figure 3E) to reduce ROS.

Acetylcholinesterase, an enzyme breaking down the neurotransmitter acetylcholine, is a feasible therapeutic target for treatment of AD (Akıncıoğlu and Gülçin, 2020). Currently AChE inhibitors such as donepezil, rivastigmine, and galantamine are commonly used to increase the level and duration of acetylcholine and facilitate cholinergic transmission in AD (Marucci et al., 2021). It has been shown that AChE activity as well as choline acetyltransferase is reduced in the cerebral cortex of patients with AD and tauopathy, indicating degenerated cholinergic neurons in both the diseases (Shinotoh et al., 2000; Hirano et al., 2006, 2018). However, the AChE activity is increased in frontotemporal dementia with parkinsonism-17 human tau transgenic mice (Silveyra et al., 2012). The findings of AChE activity in human tauopathy and the mouse model are not consistent. Our cell model that did not show significant changes in AChE activity may indicate that AChE activity is still preserved or partially impaired in this model and that inhibiting AChE activity may rescue acetylcholine levels. Among the three flavones examined, only apigenin reduced AChE activity in SH-SY5Y cells expressing proaggregator Tau (Figure 4C). Studies have shown that oxidative stress may induce AChE activity (Wang et al., 2019). However, given that all the three flavones in this study can reduce ROS, the reduced AChE activity only by apigenin may be attributed to some unknown mechanism. In the past years, intensive study efforts have been made for developing multitarget anti-Alzheimer compounds that hit several key pathogenic factors of the disease. Hybrid compounds combining a unit of potent and selective AChE inhibitor huprine Y with the 4-hydroxy-3-methoxyphenylpentanone moiety of natural antioxidant 6-shogaol have been documented (Pérez-Areales et al., 2014). In addition, herbal formulations with anticholinesterase and antioxidant activity could be benefit to memory enhancement (Nwidu et al., 2018). Being a dual antioxidant and anticholinesterase agent with tau antiaggregating property (Figures 1, 3, 4), apigenin emerges as an interesting multitarget anti-AD agent.

Upon BDNF binding, TRKB dimerized and phosphorylated to initiate intracellular signaling such as ERK, leading to activation of transcription factor CREB and downstream antiapoptotic BCL2 for neuronal survival (Walton and Dragunow, 2000). The level of phosphorylated CREB is decreased in the hippocampus of old rats with spatial memory deficits (Kudo et al., 2005; Williams et al., 2008; Xu et al., 2010). BCL2 binds to and inactivates BAX, thereby inhibiting apoptosis (Jonas, 2009). In 3 × Tg-AD mice, Tau provokes downregulation of BCL2 and increases level of BAX to lead to degeneration of cochlear spiral ganglion neurons (Wang and Wu, 2021). In our SH-SY5Y cell model, induction of proaggregated ΔK280 Tau_RD_ expression downregulated BCL2 and upregulated BAX, whereas 7,8-DHF, quercetin, and apigenin rescued changes in these gene expression (Figure 4). Although Congo red was used as a control, it did not rescue changes of p-CREB, BCL2, and BAX, which is probably due to its modest neuroprotection effect. In contrast, under the same condition, the tested flavones showed significant improvements in p-CREB, BCL2, and BAX, suggesting their promising therapeutic potential. As crosstalk between pathological Tau phosphorylation and mitochondrial dysfunction exists (Guha et al., 2020) and BCL2 counteracts the mitochondria dysfunction-mediated apoptosis, investigation of mitochondria-related apoptotic pathways through caspase-9 and caspase-3 activation may shed light on the mechanism of how quercetin and apigenin provide antiapoptotic effect on ΔK280 Tau_RD_ cells. There are still other TRKB downstream pathways such as phosphatidylinositol 3-kinase (PI3K)/AKT/mTOR. In the future, investigating if PI3K/AKT/mTOR is also involved in the neuroprotective mechanism of these flavones is warranted. Besides, although our previous study has shown the neuroprotection effects of 7,8-DHF in the primary hippocampal primary neurons and mouse models induced by Aβ (Fan et al., 2020), whether the other two flavones apigenin and quercetin also provide the similar effects either in primary neurons and mouse models should be examined in the future.

In conclusion, our results show that, in addition to 7,8-DHF, quercetin and apigenin activate TRKB signaling to upregulate downstream CREB and BCL2 expressions. In the proaggregator Tau AD cell model, these flavones improve neurite outgrowth, reduce caspase-1 and/or AChE activities by activating TRKB, as well as ameliorating ROS. As multiple pathogenic pathways are involved in AD, the potential of these flavones targeting multiple pathways may have a significant perspective for developing anti-AD drug. The effect of quercetin and apigenin as TRKB agonists should be validated in AD animal models. Assays of mitochondrial function, especially in the light of increased BCL2 expression, would provide a better connection between the reported signaling mechanisms and resulting changes in cell physiology. Also, binding of quercetin and apigenin to the TRKB receptor should be measured using surface plasmon resonance to consolidate the action as agonists of TRKB.

## Data Availability Statement

The original contributions presented in the study are included in the article/supplementary material, further inquiries can be directed to the corresponding author/s.

## Author Contributions

N-NC, T-HL, and Y-ST contributed to the execution of experiments, data analysis, and interpretation. Y-CS, K-HC, C-YL, HH-L, and M-TS contributed to review and editing. C-MC and G-JL-C contributed to the concept and design, data analysis and interpretation, obtained funding, and wrote and finalized the manuscript. All authors have read and agreed to the published version of the draft of the manuscript.

## Conflict of Interest

The authors declare that the research was conducted in the absence of any commercial or financial relationships that could be construed as a potential conflict of interest.

## Publisher’s Note

All claims expressed in this article are solely those of the authors and do not necessarily represent those of their affiliated organizations, or those of the publisher, the editors and the reviewers. Any product that may be evaluated in this article, or claim that may be made by its manufacturer, is not guaranteed or endorsed by the publisher.
